# A PPG-Based Calibration-Free Cuffless Blood Pressure Estimation Method Using Cardiovascular Dynamics

**DOI:** 10.3390/s23084145

**Published:** 2023-04-21

**Authors:** Hamed Samimi, Hilmi R. Dajani

**Affiliations:** School of Electrical Engineering and Computer Science, University of Ottawa, Ottawa, ON K1N 6N5, Canada

**Keywords:** artificial intelligence, cuffless blood pressure estimation, noninvasive blood pressure measurement, cardiovascular dynamics, deep neural network, photoplethysmogram (PPG)

## Abstract

Traditional cuff-based sphygmomanometers for measuring blood pressure can be uncomfortable and particularly unsuitable to use during sleep. A proposed alternative method uses dynamic changes in the pulse waveform over short intervals and replaces calibration with information from photoplethysmogram (PPG) morphology to provide a calibration-free approach using a single sensor. Results from 30 patients show a high correlation of 73.64% for systolic blood pressure (SBP) and 77.72% for diastolic blood pressure (DBP) between blood pressure estimated with the PPG morphology features and the calibration method. This suggests that the PPG morphology features could replace the calibration stage for a calibration-free method with similar accuracy. Applying the proposed methodology on 200 patients and testing on 25 new patients resulted in a mean error (ME) of −0.31 mmHg, a standard deviation of error (SDE) of 4.89 mmHg, a mean absolute error (MAE) of 3.32 mmHg for DBP and an ME of −4.02 mmHg, an SDE of 10.40 mmHg, and an MAE of 7.41 mmHg for SBP. These results support the potential for using a PPG signal for calibration-free cuffless blood pressure estimation and improving accuracy by adding information from cardiovascular dynamics to different methods in the cuffless blood pressure monitoring field.

## 1. Introduction

Blood pressure is the main driver of blood flow through vessels, which leads to blood circulation in the body. It also marks the health of the human cardiovascular system [1]. Hypertension, which is elevated blood pressure (BP) that results in higher force exerted on blood vessels [2], is the main risk factor for cardiovascular diseases, which, according to the World Health Organization, can often result in death and disability [3]. If untreated, hypertension may increase the risk of severe health conditions, such as myocardial infarction, stroke, atherosclerosis, blood clots, kidney failure and dementia [2]. To detect hypertension, measurement of blood pressure is necessary. In addition, because blood pressure fluctuates, it is important to perform the measurement frequently to detect any variation [4]. This variation could be caused by many factors, such as stress, food, exercise, emotion, use of medication, etc., and is usually hard to detect through infrequent clinical measurement [5]. Therefore, it is important for the general population, and especially for people with hypertension, to have regular blood pressure monitoring outside of clinical settings [6].

The measurement of blood pressure can be divided into two major methods: invasive and noninvasive. In the invasive method, a catheter is inserted into the artery to measure the blood pressure accurately. However, this method has a high risk of causing complications such as blood clotting, bleeding [7], distal ischemia, thrombosis and infection [8]. Due to the risks involved and the difficulty of the process, this method is not widely used outside of specific hospital settings [1]. On the other hand, the noninvasive methods are safe, straightforward and inexpensive, and that makes them more favorable for use both at home and in a clinic; however, this comes at the cost of lower accuracy [7].

There are different techniques for measuring blood pressure noninvasively. The gold standard for over 100 years has been the mercury sphygmomanometer. In this method a cuff placed around the upper arm is inflated to control the blood flow in the brachial artery. A trained listener is required to recognize the Korotkoff sounds through a stethoscope that is placed above the brachial artery while the cuff is deflated, easing the blood flow in the artery [9]. Another popular method that is used in home healthcare devices is ocsillometry. Oscillometry uses a cuff around the arm or the wrist and a transducer to record the pressure oscillation during the cuff deflation [9]. From the measured oscillation in blood pressure, systolic blood pressure and diastolic blood pressure are approximated based on the oscillation pattern [9].

Volume clamping and tonometry are two other noninvasive blood pressure measurement techniques. Although these methods provide continuous blood pressure values, they are mainly used in research [10]. Volume clamping uses a small cuff, that is placed on a finger, and a photoplethysmography (PPG) sensor. This method provides instantaneous blood pressure readings but is expensive, and the fact that a cuff is used in the process can make this method uncomfortable. Tonometry does not require a cuff; however, since it uses a manometer-tipped probe pressed on an artery, it is very sensitive to arm or probe movement, and therefore, requires constant calibration using a cuff-based device [10].

In recent years, cuffless blood pressure measurement methods, a generic term for blood pressure monitoring techniques that do not use a cuff, have seen increased interest due to limitations that other methods impose such as discomfort, inconvenience, high cost, complexity and lack of ability to provide continuous measurement [11]. One of the approaches that is used by many for this purpose is based on the pulse transit time (PTT), which corresponds to the time delay for a pulse wave to travel from one arterial site to another. Variations in the PTT are well-correlated with SBP and DBP [12]; however, this method requires data to be collected from two different sensors [13], which means two signals have to be monitored, pre-processed and analyzed. In addition, the two signals need to be synchronized precisely, which requires effort and has its own complications. These limitations along with the increase in use of wearable devices have given rise to research on BP monitoring methods using a single PPG signal; however, these methods are less understood in comparison to the well-established PTT-based methods [6].

The PPG signal measure changes in blood volume in capillaries by monitoring the variation in light absorption on the skin [14]. With the help of developments in PPG acquisition technology, this signal can be used to estimate BP noninvasively and continuously [15]. In this method, blood pressure is not measured, but rather it is predicted based on analysis of the waveform [5]. Studies have shown that there are similarities between PPG and the arterial blood pressure (ABP) waveform in terms of morphology [16] and dynamics [17], and therefore, extracted features from PPG will carry valuable information regarding the BP [16]. Features that are mostly generated from pulse morphology within individual beats are most commonly used in various machine learning models to estimate blood pressure [11]. Sulochana [18] provides a recent review of PPG-based methods that have been used in different studies. The methods can be a variation of linear regression models, such as the work by [19] and [16], or based on different approaches of artificial neural networks (ANN) [20,21,22]. One of the derivatives of the ANN that has gained popularity in recent years is deep learning [23]. Different approaches within this method could include recurrent neural network (RNN), long short-term memory (LSTM) and the gated recurrent unit (GRU) that was used by [10], convolutional neural network (CNN), or the combination of CNN and LSTM that was used by [23], or some new approaches with modified versions of deep learning such as deeply supervised net (DSN) used by [24] and U-NET that was the basis for the research conducted in [25]. These methods have gained attention due to their often superior performance compared to traditional ANN.

In previous work, we investigated BP estimation based on information from dynamic changes in PPG signal while a mathematical model was used for calibration stage [11]. In this work, we investigated the possibility of replacing the calibration stage with information from PPG morphology in order to provide a calibration-free method for BP estimation. To implement this process, we estimated BP by using both a mathematical model and PPG morphology, then along with features of cardiovascular dynamics expressed in the PPG oscillation, BP values were estimated and compared to reference measurements. In addition to the possibility of achieving a calibration-free estimate, this approach was taken because there is a high possibility that the dynamics of human cardiovascular system carry important information related to BP, while features derived solely from the morphology of individual pulses have not provided a sufficient level of accuracy in BP estimation [26].

In this work, we used mean point-to-point (mPTP) features derived from the morphology of the PPG pulses and averaged over all the pulses for the duration of the signal to develop our estimation model. This is a novel approach in BP estimation based on PPG morphological characteristics. Another innovation in this work is the use of PPG morphology features along with the information from the dynamics of these signals. Furthermore, to the best of our knowledge, there has been no previous research conducted on replacing the calibration stage in the BP estimation process with the PPG morphology features.

## 2. Materials and Methods

**Figure 1:**
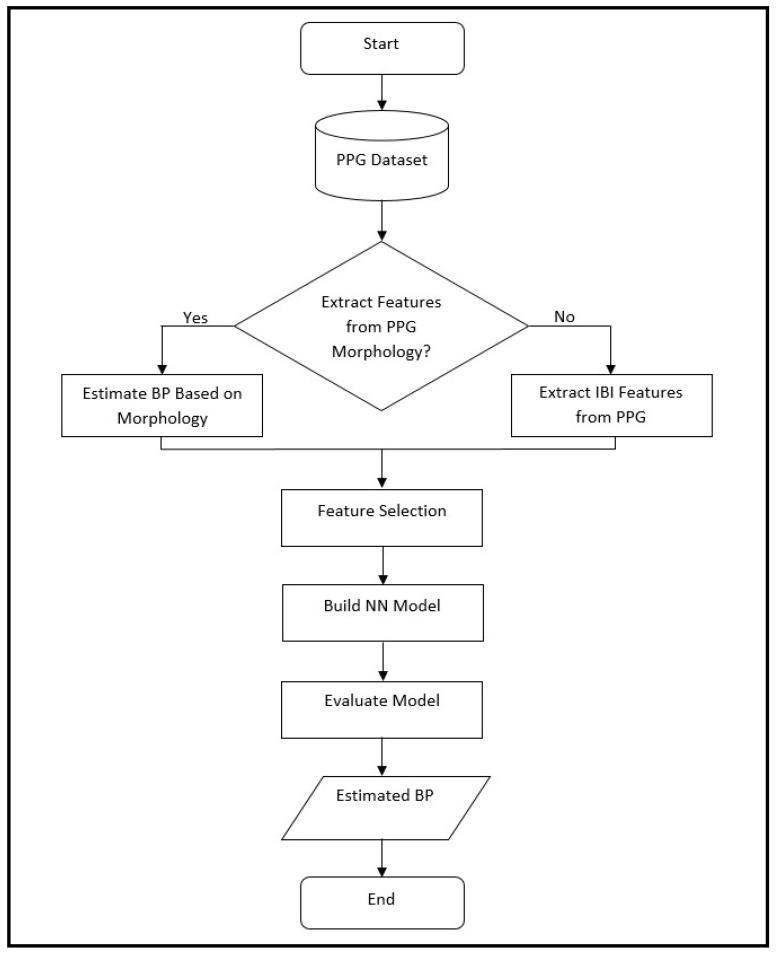
Flowchart of the proposed method in this paper for cuffless blood pressure estimation, which shows the process of BP estimation for design, evaluation and testing. During the design phase, the ANN model is based on leave-one-out (LOO) method, where it is evaluated on data from a single patient at a time and repeated over all patients. The same structure is used during the final testing phase where the ANN is trained on the complete dataset, and then, tested on a new set of data from 25 patients that have never been seen by the structure. In this process the choice of whether to use extracted features from PPG morphology depends on whether the analysis is based on morphology or interbeat interval (IBI) features (as explained in the text).

Figure 1 shows a high-level flowchart for the proposed methodology for cuffless blood pressure estimation, which uses information related to cardiovascular dynamics and morphology, both derived from a PPG signal recorded with a single sensor. Information related to cardiovascular dynamics is extracted based on our previous work [11], and the ones related to PPG morphology are inspired by the proposed methodologies in two previous studies [10,20]. This figure shows a general design that is used during the design development and evaluation, as well as the testing phase with new data. During the development of the ANN and evaluation process, the leave-one-out method (LOO) is used, where each iteration of LOO takes one patient with all associated signals out for testing, while it trains on the remaining signals. In the testing phase, the ANN is trained on a complete dataset and tested on signals from 25 new patients that have never been seen by the structure.

We can divide our work into two major parts based on the PPG morphology feature extraction, where one uses a feedforward artificial neural network model and the other uses a deep learning model (Figure 1).

### 2.1. Bio-Signal Datasets

In this study, we used two separate bio-signal datasets, one from the University of Queensland Vital Signs Dataset [27] and another from the University of California Irvine (UCI) Machine Learning Repository [28].

Data from the University of Queensland includes ECG, PPG and noninvasive arterial BP waveforms recorded from 32 patients (25 under general anesthetics, 3 under spinal anesthetics, and 4 sedation cases) in four operating rooms, and sampled at the rate of 100 Hz. The recording ranged from 13 min to 5 h with a median of 105 min. These data cover the complete duration of the anesthesia that patients go through, which means there could be rapid variations in vital signs in the induction and emergence phases of anesthesia and surgery. This makes the dataset unique compared to others, such as data collected from intensive care units [27].

The second dataset, the one from UCI, is a subset of the Multiparameter Intelligent Monitoring in Intensive Care (MIMIC II) data. MIMIC II is an open source multiparameter recording provided by the PhysioNet organization that is collected from 15,000 intensive care units (ICU) in multiple hospitals. The collected signals are sampled at 125 Hz with at least 8-bit resolution. The following process was followed in order to generate the subset of the MIMIC II dataset that is provided by UCI:

- The original data are divided into fixed size signal blocks. Each block is processed through a simple averaging filter to smoothen the signal;
- Any block with irregular blood pressure value or heart rate is removed;
- Autocorrelation is calculated for PPG signal to indicate the degree of similarity between successive pulses in a block;
- Any block with high alteration between successive pulses, based on the calculated autocorrelation in the previous step, is removed [28].

In our work, we used PPG signals and noninvasive arterial BP signals from the University of Queensland data, and PPG signals and ABP signals from the UCI data. In the case of ABP, the reference values for systolic blood pressure (SBP) and diastolic blood pressure (DBP) were calculated by averaging the values of the peaks and troughs of the waveform (respectively) for each patient over the duration of the test signal. For the data from the University of Queensland, the reference values were obtained by averaging the provided beat-to-beat noninvasive SBP and DBP values over the duration of the signal for each patient. In both cases we visually inspected the waveforms and chose the ones with minimal or no interruption. In the case of data from UCI, since the dataset comprises some signals with short duration, we only selected the ones with 8 to 10 min in length and excluded the signals that were short in duration. The resulting datasets consisted of signals from 200 patients for the data from UCI and 30 patients for the data from the University of Queensland. In addition, we set aside an extra set of signals, 25 randomly selected patients from the UCI dataset with an SBP range of 105.40 mmHg to 151.70 mmHg and a DBP range of 53.85 mmHg to 72.93 mmHg, to be used as never-seen test data in the final step of our work.

### 2.2. Morphology-Based Estimation

**Figure 2:**
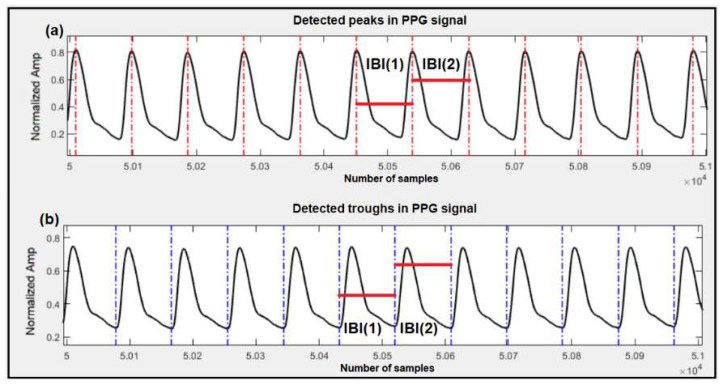
Detection of peaks, troughs and corresponding IBIs in the PPG signal. (**a**) Detected peaks are marked with vertical red lines and sample IBIs, used for SBP estimation, are marked with horizontal red lines; (**b**) Detected troughs are marked with vertical blue lines and sample IBIs, used for DBP estimation, are marked with horizontal red lines.

For this part, we used both datasets mentioned earlier to estimate SBP and DBP. These are the same datasets that we used in our previous works [11,17], and by reusing them in this research (that proposes a different method), the estimation results and accuracy of the models can be compared. Our work has mainly focused on features related to human cardiovascular dynamics. In order to extract dynamics features, we used a modified version of Pan and Tompkins’ QRS detection algorithm to detect the peaks and troughs of the PPG signals and ultimately calculate the interbeat intervals (IBI) in the signal under test. Figure 2 shows a sample of peak and trough detection for the PPG signal using the algorithm and the resulting IBIs. We used IBIs of the peaks for SBP estimation, and IBIs of the troughs for DBP estimation. The details of this process are reported in our previous work [11].

In [17], we used the dataset from UCI and extracted features from the IBI series of the PPG signals through the time domain, frequency domain, and nonlinear analysis, as well as the features of pulse pressure, systolic and diastolic waveforms, and baroreflex sensitivity. After a feature selection process through wrapper subset evaluation with the forward greedy stepwise search [29], we ended up using 5 features for SBP estimation (mean IBI, NNx, pNNx, SD2 and α1) and 6 features for DBP estimation (mean IBI, NNx, pNNx, PRVTi, SampleEn and IBI ratio of LF/HF). The details of the methodology and calculation of each of these features are reported in [17].

In [11], we used the dataset from the University of Queensland to extract cardiovascular dynamics features. In that work, we investigated the possibility of BP estimation using calibration-free and calibrated cardiovascular dynamics obtained with the PPG. The IBI features selected for the calibration-free method were NNx, α1, LF and HF for the SBP estimation, and SDNN, RMSSD, SD1, LF and α1 for the DBP estimation. For the calibrated model, the selected IBI-related features were SampleEn and α1 for the SBP estimation and SampleEn and HF for the DBP estimation. To the features for the calibrated method, we also added the estimation from a calibrated mathematical model as an extra feature for both SBP and DBP.

In this work, blood pressure was estimated based on the same features, as mentioned above (for both datasets), when the same methodology was required for result comparison.

#### 2.2.1. Feedforward Artificial Neural Network Model

In this work, BP estimation from the PPG morphology was used in addition to extracted features from the PPG dynamics. For this purpose, 21 features were extracted based on the research conducted in [20]. However, unlike [20], which collects the features in the context of one point-to-point (oPTP), we used the mPTP method, which takes an average of the feature over an interval of the signal. This modification was made because previous studies on BP estimation have shown that the oPTP method is not robust, and one way to overcome this shortcoming is to use the mPTP technique [30].

**Figure 3:**
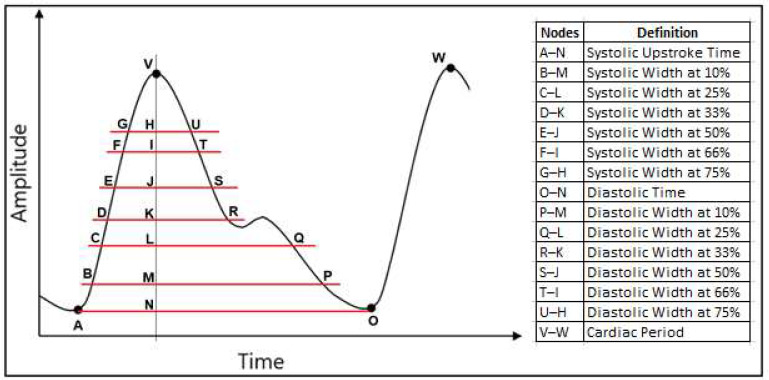
Extracted parameters from the PPG signal to generate the 21 features used in this study.

The extracted features for the estimation of SBP and DBP are listed below and shown in Figure 3.

- Cardiac period;
- Systolic upstroke time;
- Diastolic time;
- Diastolic width at 10%, 25%, 33%, 50%, 66% and 75% of the pulse height;

For example, the diastolic width at 10% is calculated based on the time difference between the point at 10% of the peak amplitude that coincides in time with the peak (point M in Figure 3) and the point at 10% of the peak amplitude located in the diastolic region of the pulse (point P in Figure 3).

- Sum of systolic width and diastolic width at 10%, 25%, 33%, 50%, 66% and 75% of the pulse height;
- Ratio of diastolic width to systolic width at 10%, 25%, 33%, 50%, 66% and 75% of the pulse height.

As for the structure of the artificial neural network, a multilayer feedforward back propagation ANN with 21 inputs and 2 output neurons was considered to simultaneously estimate SBP and DBP. In this architecture, 2 hidden layers were included with 35 neurons on the first hidden layer and 20 on the second one. The hidden layer neurons have sigmoid activation functions, while the output layer neurons for both SBP and DBP possess linear activation functions. This network structure was implemented based on [20].

#### 2.2.2. Deep Learning Model

In this part we looked into ANN deep learning for BP estimation based on the morphology features of the PPG. Seven parameters were selected based on the work in [10], and the mPTP method was used to generate each of the features that are listed below.

- Cardiac period;
- Diastolic time;
- Diastolic width at 25% and 75% of the pulse height;
- Sum of systolic width and diastolic width at 33% and 75% of the pulse height;
- Ratio of diastolic width to systolic width at 10% of the pulse height.

For the ANN model, we considered three different approaches: feedforward deep neural network, LSTM and GRU [10]. The structure of each model is described below.

- Feedforward deep neural network: This model is similar to the one used in Section 2.2.1, which consisted of non-recurrent feedforward connections between the neurons, and it was constructed with three hidden layers containing 70, 100 and 150 neurons for Layers 1, 2 and 3 respectively [10];
- LSTM: Long short-term memory uses feedback connections to process sequential time domain data. It was originally developed to overcome the vanishing gradient problem during the training of the recurrent neural network due to long term prediction [10]. The LSTM used in this work was constructed with two hidden layers of 64 and 512 neurons;
- GRU: The gated recurrent unit is similar to the LSTM but since it uses fewer parameters, it is somewhat less computationally expensive. It has also shown better performance on certain smaller datasets compared to LSTM [10]. The network used here was constructed with three hidden layers of 128, 256 and 512 neurons in consecutive layers.

In the deep learning models, a sigmoid activation function is used for the feedforward deep neural network, while a rectified linear unit (ReLU) activation function is used for both LSTM and GRU approaches.

### 2.3. Blood Pressure Estimation Model

The blood pressure estimation result from the PPG morphology based on the above-mentioned methodologies was considered an extra feature to be added to the ones extracted from cardiovascular dynamics. The dataset was partitioned based on the leave-one-out method, where all features from one patient were set aside to be used as the test data, while the remaining features were split into 85% training and 15% validation. This process was repeated for all patients to cover the entire dataset. The purpose of setting aside 15% of the dataset for validation was to use the early stopping technique in order to avoid overfitting [31].

To estimate both systolic and diastolic blood pressure, an ANN with a two-layer feedforward network structure was used for regression. The two layers consisted of a sigmoid layer followed by a linear output layer. Based on our previous work with the same datasets [11,17] and the performance of the model with different numbers of neurons, a hidden layer with ten neurons was selected in this study. The Bayesian regularization backpropagation algorithm was used to train the network, and the structure was fixed prior to applying it to the test data.

The estimation performance was evaluated based on the mean error (ME), the mean absolute error (MAE), and the standard deviation of error (SDE) obtained with the test data. The ME and MAE are calculated using the following equations:

(1) $$ME=\frac{\sum_{i=1}^{n}y_{i}-x_{i}}{n},$$

(2) $$MAE=\frac{\sum_{i=1}^{n}\left|y_{i}-x_{i}\right|}{n},$$

where $y_{i}$ is the prediction and $x_{i}$ is the true value for the blood pressure from the dataset. The true values are determined by averaging either the noninvasive BP (for the data from the University of Queensland) or the invasive arterial BP (for the data from UCI) of each patient over the duration of the test signal.

The SDE was calculated using the following equation:

(3) $$SDE=\sqrt{\frac{\sum_{i=1}^{n}{\left|e_{i}-\overset{-}{e}\right|}^{2}}{n}},$$

where $e_{i}$ is the error between the prediction and the true value ($e_{i}=y_{i}-x_{i}$) for each estimation, and $\overset{-}{e}$ is the average of $e_{i}$.

## 3. Results

The BP estimation results in this paper are based on three different datasets: PPG and noninvasive BP from 30 patients (University of Queensland dataset), the PPG and invasive arterial blood pressure waveform from 200 patients (UCI dataset) and the PPG and invasive arterial blood pressure waveform from 25 new patients (UCI dataset).

### 3.1. Estimation of BP with 30 Patients from the University of Queensland Dataset

In this part, we estimated both systolic and diastolic blood pressure from 30 patients using the following three methods:

- Features from cardiovascular dynamics extracted from PPG signal. This is a calibration-free method that we developed in [17];
- Information based on PPG morphology features. Estimation for both SBP and DBP was performed based on 21 extracted morphology features;
- A calibrated mathematical model. This is part of our previous work [11], where we used a mathematical model to calibrate the blood pressure estimator.

**Figure 4:**
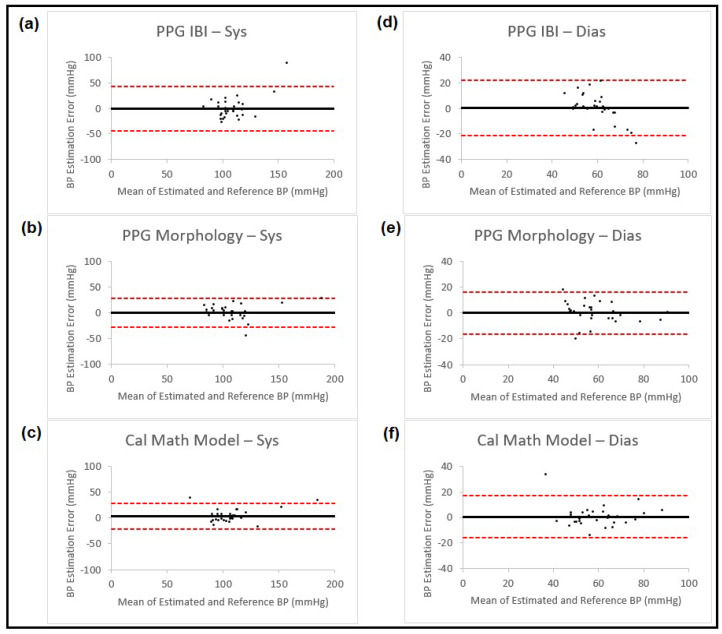
Bland–Altman plots presenting systolic and diastolic blood pressure estimation using three different methods (IBI features from PPG, PPG morphology features and calibrated mathematical model). The left column shows the results for systolic BP and the right column for diastolic BP. Each of the plots is named corresponding to the method that is used for estimation. In all the plots, the black horizontal line is the bias and the two dashed red lines are the 95% limits of agreement.

Results from the proposed methods are shown in Table 1, and the corresponding Bland–Altman plots for these estimations are shown in Figure 4.

We also combined the estimation methodologies mentioned above to obtain higher accuracy. This was completed through two different approaches:

- Fusion technique;
- Feature combination.

In fusion technique, we used the sensor fusion method to combine different estimation results. Sensor fusion is a process used to combine data derived from different sources in order to reduce uncertainty that would otherwise have come from each of the sources, and it is calculated using [32]

(4) $$x_{3}=\sigma_{3}^{2}(\sigma_{1}^{-2}x_{1}+\sigma_{2}^{-2}x_{2}),$$

where $x_{3}$ is the resulting information and $x_{1}$ and $x_{2}$ are the measurements obtained from two sources with noise variances $\sigma_{1}^{2}$ and $\sigma_{2}^{2}$, respectively. $\sigma_{3}^{2}$ is the variance of the combined estimate and can be calculated as

(5) $$\sigma_{3}^{2}={\left(\sigma_{1}^{-2}+\sigma_{2}^{-2}\right)}^{-1},$$

**Figure 5:**
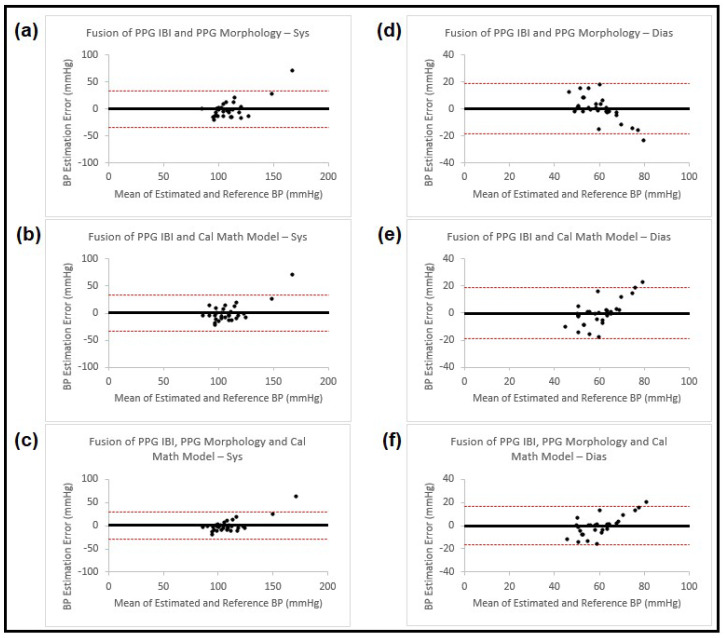
Bland–Altman plots presenting systolic and diastolic blood pressure estimation using fusion on three different methods (IBI features from PPG, PPG morphology features and calibrated mathematical model). The left column (**a**–**c**) shows the results for systolic BP, and the right column (**d**–**f**) for diastolic BP. Each of the plots is named accordingly. In all the plots the black horizontal line is the bias and the two dashed red lines are the 95% limits of agreement.

Table 2 shows the estimation results for both SBP and DBP obtained using the sensor fusion process on the results achieved from earlier-mentioned methods, and Figure 5 presents the corresponding Bland–Altman plots for the estimation results.

**Figure 6:**
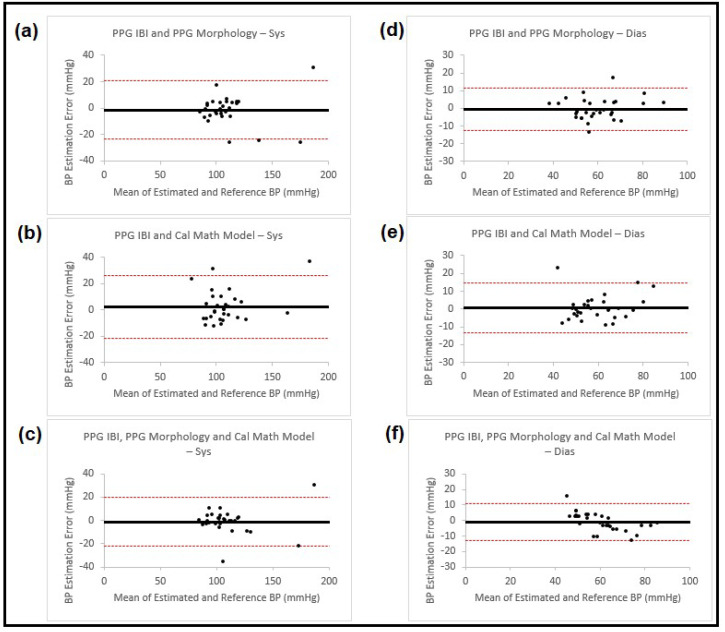
Bland–Altman plots presenting systolic and diastolic blood pressure estimation using three different sets of features from PPG waveforms (IBI and morphology, IBI and calibrated mathematical model and IBI, morphology and calibrated mathematical model). The left column (**a**–**c**) shows the results for systolic BP and the right column (**d**–**f**) for diastolic BP. Each of the plots is named accordingly. In all the plots, the black horizontal line is the bias and the two dashed red lines are the 95% limits of agreement.

In the second method, we used blood pressure estimation from PPG morphology or the calibrated mathematical model as extra features and added them to the PPG IBIs, one at a time or both together (having three combinations that were the same as the ones in the fusion process). We then used wrapper subset evaluation with the forward greedy stepwise search method and determined the best features for the ANN estimator. The blood pressure estimation results for SBP and DBP obtained using this method are shown in Table 3 and the corresponding Bland–Altman plots for the estimation results are shown in Figure 6.

Comparing the two previously mentioned methods (fusion process and feature combination) shows that the fusion process resulted in lower accuracy. As for the results obtained from combining PPG dynamics features and estimation from PPG morphology and the calibrated mathematical model, they showed that while adding either results from morphology or calibration to the PPG IBI features improved the estimation results, adding both at the same time did not have much of an improvement, especially for diastolic blood pressure. Considering this new finding, we decided to compare the correlations between the estimation results obtained using PPG dynamics, PPG morphology and the PPG mathematical calibration model by calculating the correlation coefficient:

(6) $$\rho_{X,Y}=\frac{cov(X,Y)}{\sigma_{X}\sigma_{Y}},$$

where *cov* is covariance, $\sigma_{X}$ is the standard deviation of *X*, and $\sigma_{Y}$ is the standard deviation of *Y*.

**Figure 7:**
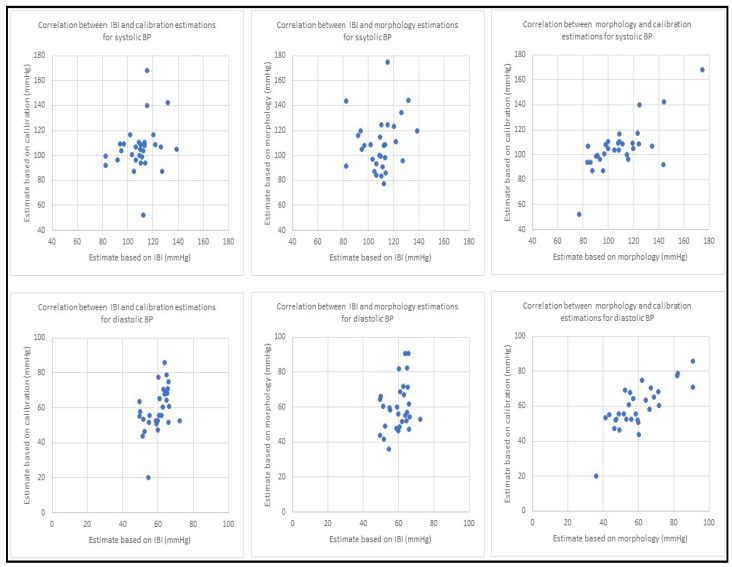
Scatter diagrams for blood pressure estimation using three different methods. The top row has the results for systolic BP and bottom row shows the results from diastolic BP. For both rows, the first plot from the left shows correlation between BP estimation using PPG dynamics and PPG morphology, the second plot displays BP estimation correlation between PPG dynamics and the calibration model and the third plot illustrates blood pressure estimation correlation using morphology and the calibration model. All the results are based on collected PPG waveforms from 30 patients. All estimates in this figure are in mmHg.

The resulting correlation coefficients showed that while there is a low level of correlation between the estimation results from IBI and morphology or IBI and the calibration method, the blood pressure estimation values from morphology and calibration methods are highly correlated. Correlation results were similar for both SBP and DBP. These correlations are shown in Table 4 and the scatter diagrams are presented in Figure 7.

Given the finding of high correlation between BP estimation results obtained from PPG morphology features and the calibration model, we used the estimation from the PPG morphology features as a substitute for the calibration model in our remaining experiments.

### 3.2. Estimation of BP with 200 Patients from the UCI Dataset

**Figure 8:**
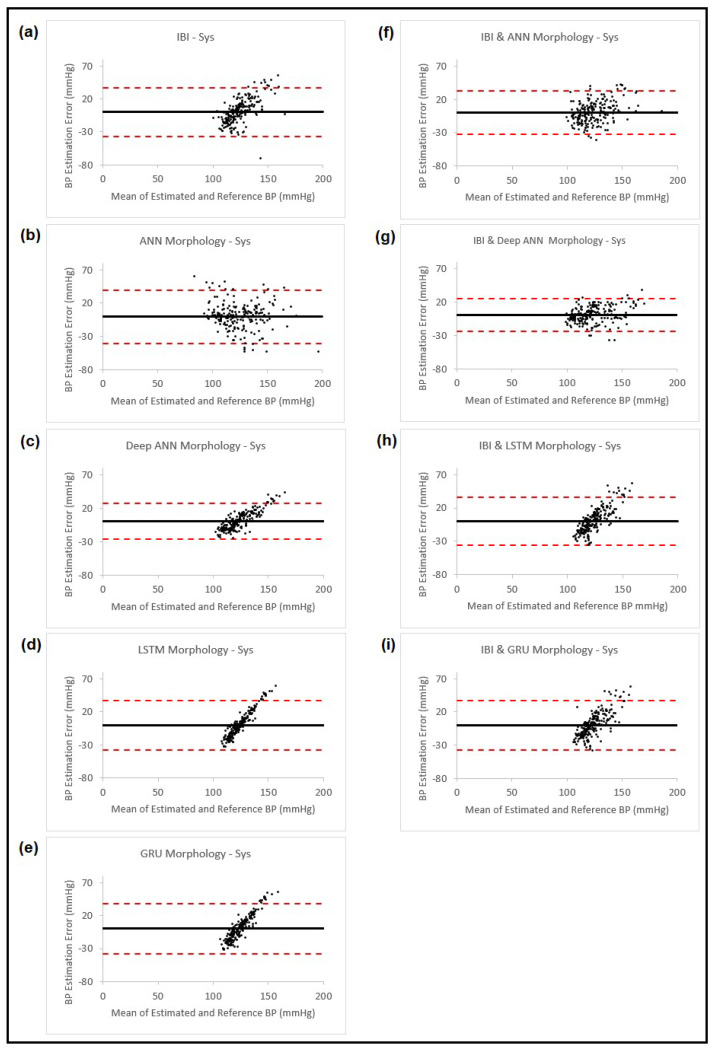
Bland–Altman plots presenting systolic blood pressure estimation using different methods: (**a**) Estimation using PPG dynamics features and ANN model; (**b**) Estimation using PPG morphology features and ANN model; (**c**) Estimation using PPG morphology features and feedforward deep NN model; (**d**) Estimation using PPG morphology features and LSTM model; (**e**) Estimation using PPG morphology features and GRU model; (**f**) Estimation combining the estimated BP result from (**b**) and PPG dynamics and use them in ANN model; (**g**) Estimation combining the estimated BP result from (**c**) and PPG dynamics and use them in ANN model; (**h**) Estimation combining the estimated BP result from (**d**) and PPG dynamics and use them in ANN model; (**i**) Estimation combining the estimated BP result from (**e**) and PPG dynamics and use them in ANN model. In all the plots, the black horizontal line is the bias and the two dashed red lines are the 95% limits of agreement.

**Figure 9:**
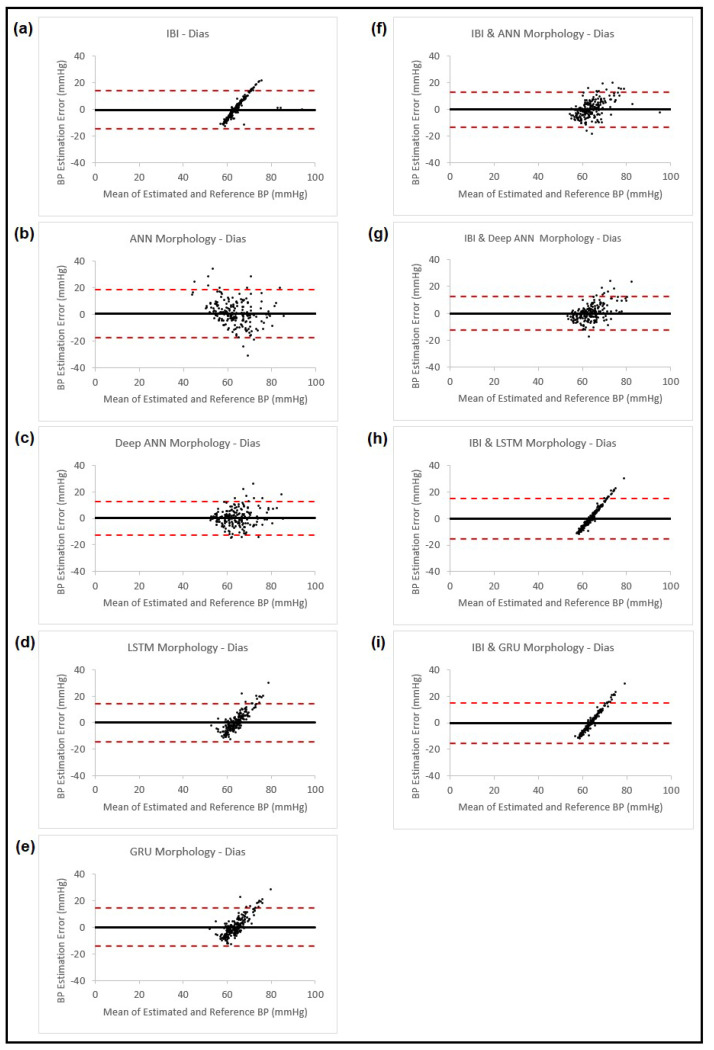
Bland–Altman plots presenting diastolic blood pressure estimation using different methods: (**a**) Estimation using PPG dynamics features and ANN model; (**b**) Estimation using PPG morphology features and ANN model; (**c**) Estimation using PPG morphology features and feedforward deep NN model; (**d**) Estimation using PPG morphology features and LSTM model; (**e**) Estimation using PPG morphology features and GRU model; (**f**) Estimation combining the estimated BP result from (**b**) and PPG dynamics and use them in ANN model; (**g**) Estimation combining the estimated BP result from (**c**) and PPG dynamics and use them in ANN model; (**h**) Estimation combining the estimated BP result from (**d**) and PPG dynamics and use them in ANN model; (**i**) Estimation combining the estimated BP result from (**e**) and PPG dynamics and use them in ANN model. In all the plots, the black horizontal line is the bias and the two dashed red lines are the 95% limits of agreement.

In this part, we estimated both the systolic and diastolic blood pressure of 200 patients using features from cardiovascular dynamics and PPG morphology. As was mentioned in Section 2.3 above, the blood pressure was estimated using features from the PPG morphology, and the estimated value was added to the collected features from cardiovascular dynamics to replace the calibration step we had used in our previous study [11]. The BP estimation based on the PPG morphology features was carried out using the following four different ANN models: the feedforward back propagation neural network, the feedforward deep neural network, LSTM and GRU. The blood pressure estimation results using cardiovascular dynamics through PPG IBI, the different models for the PPG morphology, and the proposed method obtained by using estimation using the PPG morphology in addition to the PPG IBI, are shown in Table 5, the corresponding Bland–Altman plots for SBP are presented in Figure 8 and for DBP, in Figure 9.

### 3.3. Estimation of BP with 25 New Patients from the UCI Dataset

**Figure 10:**
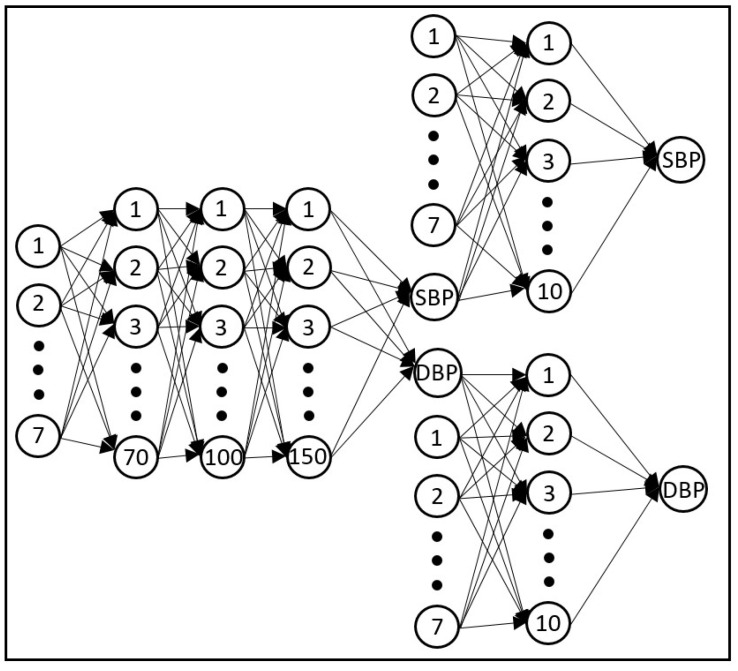
Structure of the proposed neural network model. This network consists of three subnetworks. The one on the left has seven inputs that are PPG morphology features, three hidden layers that use sigmoid activation functions and an output layer that uses a linear activation function. The outputs of this subnetwork are estimated SBP and DBP that are then used in the subsequent subnetworks to estimate the final BP values. Each of these two subnetworks (on the right) has eight inputs (seven features related to cardiovascular dynamics plus the estimated BP value from morphology features from the prior subnetwork). Furthermore, each of these two networks has a single hidden layer with a sigmoid activation function and an output layer with a linear activation function. The resulting SBP and DBP from this stage are the final BP estimation values.

In this part, we tested our proposed estimation model with the data from 25 patients that were not seen by the model before. Our feature set for the proposed ANN model consisted of the seven PPG dynamics features (SDNN, PRVTi, TINN, LF, HF, α1 and α2) and the BP estimation obtained by using the PPG morphology features with feedforward deep neural network. Figure 10 is a graphical representation of the structure of the proposed model.

**Figure 11:**
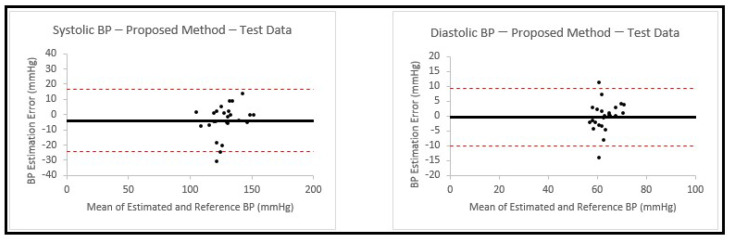
Bland–Altman plots presenting systolic and diastolic blood pressure estimations using the proposed model on the test data from 25 never-seen-before patients. In both plots, the black horizontal line is the bias and the two dashed red lines are the 95% limits of agreement.

This model was chosen based on the results achieved from earlier experiments in Section 3.2, which showed that the feedforward deep neural network model gave the best performance among the methods that were used. This allowed us to have a calibration-free model that can be used to estimate both SBP and DBP. We then trained the model on 200 signals that were used in Section 3.2. The trained model was used to estimate blood pressure values for the 25 new patients. The estimation results and the related errors are shown in Table 6, with the corresponding Bland–Altman plots shown in Figure 11.

## 4. Discussion

The results of this study indicate that the proposed method based on PPG dynamics over short intervals combined with the blood pressure estimation from the PPG morphology could be used as a calibration-free technique to obtain blood pressure estimation using only a single photoplethysmogram signal. Although reference BP values were used during the process of training the neural network, this method is considered calibration-free since no calibration is involved in the actual usage when testing on new subjects. One advantage of this method is that the PPG dynamics, which are obtained only from timing variation between the peaks and troughs of the signal and that are used as part of the model, would be expected to be less susceptible to poor sensor placement or different skin colors than the commonly used pure morphology features. This may be one of the reasons why we see improvement in estimation when IBI features are added to the estimation from morphology.

In the first part of this study, we investigated a calibrated model and explored the possibility of replacing the calibration step with estimation from morphology features to obtain a calibration-free method. We started from our previous study [11], where we extracted 16 features from PPG IBI, and through wrapper subset evaluation with a forward greedy stepwise search technique, we selected four features for SBP estimation and five features for DPB estimation. Using the same feature selection method for the calibrated model resulted in choosing two IBI-related features for both SBP and DBP estimations in addition to the calibration feature. To study the possibility of replacing the calibration step with information from the PPG morphology, we then replaced the calibration feature with an estimation obtained from the morphology. Our results showed that similar BP estimation accuracy was obtained from the following three different methods: a calibrated IBI model, replacing the calibration feature with a morphology estimation in the calibrated IBI model, or adding a PPG morphology estimation to the IBI calibrated model. While adding calibration or estimation from morphology led to substantial reduction in most of the error measures compared to the estimation from the IBI features, particularly the critical measures of SDE and MAE (by about 50% in both cases), when they were both added at the same time, the improvement in error measurement (compared to when only estimation from morphology was added to the IBI) were minimal (SDE and MAE were reduced by 5% and 1% for SBP and 1% and 1% for DBP, respectively). To further evaluate the possibility of replacing calibration with estimation from the PPG morphology, we looked at the correlation between blood pressure estimation when IBI features, PPG morphology features or mathematical estimation model were used. The results showed that while there were no strong correlations between the estimation results from IBI and the calibrated mathematical model or IBI and PPG morphology, the blood pressure estimation from the PPG morphology features and the calibrated mathematical model were relatively strongly correlated (correlation coefficient equal to 0.74 for SBP and 0.78 for DBP). Therefore, this suggested that the calibration step may be replaced by estimation from the PPG morphology (when using our dataset and the proposed methodology), which results in a calibration-free estimation method. Our experimental results also showed that the PPG dynamics can be added to the estimation from the PPG morphology to increase the overall estimation accuracy.

In the second part of the work, we looked at improving the estimation accuracy of the proposed method and also confirming that the information from the PPG dynamics could improve the estimation results obtained from different methods. For this purpose, we based the work on our previous study of BP estimation using a calibration-free method [17], where we extracted 16 IBI features from PPG signals and selected 5 features for SBP estimation and 6 features for DBP estimation, based on the same feature selection technique that was used in the first part. We obtained the estimation from the PPG morphology (21 features) with a feedforward back propagation artificial neural network. This was the same methodology that we used in the first part of this study. We also proceeded with implementing three different deep learning methods (feedforward deep NN, LSTM and GRU) to be used with seven features from the PPG morphology for the BP estimation to improve the accuracy of our proposed model. The estimation results, in particular the measures of SDE and MAE, were compared for the five different methods (IBI features with ANN, morphology features with ANN, morphology features with three deep learning models). We then added the obtained estimation from the PPG morphology (through different models) to the PPG IBI features (same as the proposed calibration-free method in part one) and selected eight features (seven IBI features and the PPG morphology estimate) for both SBP and DBP using the wrapper subset evaluation with a forward greedy stepwise search technique. The eight resulting features were used in an ANN model to estimate the finalized estimation for SBP and DBP. The results showed that for all four models, adding IBI features improved the accuracy. This confirms that PPG dynamics contain useful information that can improve blood pressure estimation for different methods. We also found that using the proposed feedforward deep NN model consisting of more hidden layers and neurons instead of the shallow NN that was originally used in our proposed method, reduced the measured error; particularly, the critical measures of SDE and MAE by 24% and 25% for SBP and 6% and 13% for DBP.

In the last part of this work, we used the proposed model to estimate SBP and DBP with PPG signals from 25 new patients. This part was important in validating our method since the information from these patients was not previously seen by our model. The result showed a SDE and MAE of 10.4 mmHg and 7.41 mmHg for SBP, and 4.89 mmHg and 3.32 mmHg for DBP. This result presents an improvement in accuracy when compared to our previous studies.

In this work, we used two different datasets: one was the University of Queensland Vital Signs Dataset [27], from which we extracted PPG and noninvasive arterial BP waveforms, and the other dataset was derived from the University of California Irvine Machine Learning Repository [28], from which we used PPG signals and invasive arterial blood pressure signals. Except for the 25 new patients, who did not take part in any of our previous studies, these are the same datasets and signals that were used in our previous studies, [11] and [17], which resulted in different IBI-related features being selected for blood pressure estimations in these two studies. In our current work, with the new proposed method for BP estimation, we also observed similar discrepancy when it came to IBI feature selection. As was discussed in [11], this difference could be the result of different target data for the two datasets (noninvasive BP for the University of Queensland data and invasive BP for UCI data), as there are differences in BP readings between invasive and noninvasive methods [33]. The population of the datasets could also have an impact on the feature selection. The data provided by UCI were collected in intensive care units, where the patients were possibly under the influence of medications that could impact blood pressure dynamics or, in the case of inotropes, could result in a different BP measurement between invasive and noninvasive methods, with variations being directly related to the amount of medication that was used [34]. On the other hand, the data from the University of Queensland were collected from patients undergoing anesthesia for surgery, which could result in rapid and dynamic vital sign changes during induction and emergence phases of anesthesia.

Several of the Bland–Altman plots show an increasing (or in some cases a decreasing) trend in the error, with increase in the mean of the BP estimated using our models and the reference BP. A similar effect has been found in the past in Bland–Altman analyses of BP pressure estimated using other methods [35,36]. The study by Shimada et al. [35] carefully examined this effect and determined that it was likely due to differences in interindividual variability obtained with the two methods that were compared, which may also apply to our study. However, it should be noted that in our final structure, we used a model that did not show severe trends for SBP and DBP of this type (Figure 11).

The lower errors found for DBP vs. SBP also follow the same trend that we found with different methods, and it could be due to the strong relation between HRV indices and both SBP and DBP in females, while this relation is limited to DBP for males [37]. The datasets that we used in this study did not include information regarding gender composition. If we assume that the male and female participants were close in number, then the BP estimation result based on IBI dynamics will show lower accuracy for SBP than DBP, and that is what we saw in this study. Additionally, since changes in IBI are more clearly present in DBP compared to SBP [38], this could be a contributor to the higher estimation accuracy of DBP. Another possible reason behind the lower reported error for DBP could be because the baseline DBP values are lower compared to the ones for SBP, which could also cause the errors to be lower.

This study also had some limitations. First, the datasets did not include some useful information regarding characteristics such as race, age, gender and weight that have an impact on blood pressure levels and that could provide valuable information contributing to the accuracy of the estimation results [39,40]. Second, the sizes of the datasets could have impacted the deep learning algorithm results. Since the effectiveness of deep learning techniques is impacted by the size of the dataset [41], having a larger dataset ultimately could improve the blood pressure estimation results. Third, in this work, the quality of the PPG signals was inspected manually, and like any other non-automated process, they could introduce errors, especially with the sizes of the dataset that were analyzed. Implementing an automated approach for this process would be highly beneficial for real-life scenarios. Finally, our replacement of calibration with estimation based on morphology was evaluated on a relatively limited dataset (University of Queensland dataset from 30 patients) with short measurement intervals (less than 5 h). The ability to retain a calibration-free estimation needs to be confirmed by testing longer signal durations over more samples.

## 5. Conclusions

In this paper, we focused on a cuffless blood pressure estimation based solely on information carried by PPG signals. We demonstrated that the cardiovascular dynamics carry useful information that can help to improve the accuracy of BP estimation when features based on pulse morphology are also used. Another finding of this work was that we were able to replace a calibration method (that was introduced in our previous work [11]) with an estimation from the PPG morphology, and by doing so, we were able to provide a calibration-free method for blood pressure estimation, which provided similar accuracy as the calibrated method. It is worth mentioning that the estimation made using PPG morphology was reliant on the same features used in previous studies [10,20], and did not incorporate any new ones. However, if different or additional PPG morphology features are used, there is a possibility to further improve the overall estimation accuracy.

Since in this paper adding information from cardiovascular dynamics to a number of different BP estimation techniques based on pulse morphology showed considerable improvement in the estimation accuracy, this approach may also be useful for improving the BP estimation accuracy of other methods, such as the widely used ones that are based on the pulse transit time.

## Figures and Tables

**Table 1:** Systolic and diastolic blood pressure estimation performance from 30 patients using: (1) PPG dynamics features, (2) PPG morphology features, and (3) Calibrated mathematical model, as explained in the text. Results are averaged over the 30 patients.

	Systolic BP (mmHg)			Diastolic BP (mmHg)		
	ME	SDE	MAE	ME	SDE	MAE
PPG IBI	−0.39	22.16	15.26	0.14	10.97	7.54
PPG Morphology	0.06	14.22	10.10	0.01	8.32	6.16
Calibrated Mathematical Model	3.18	12.49	9.11	0.45	8.36	5.47

**Table 2:** Blood pressure estimation performance using fusion process on the results obtained from: (1) IBI features of PPG signals and PPG morphology features; (2) IBI features of PPG signals and calibrated mathematical model; and (3) IBI features of PPG signals, PPG morphology features and calibrated mathematical model from 30 patients. Results are averaged over all the patients.

	Systolic BP (mmHg)			Diastolic BP (mmHg)		
	ME	SDE	MAE	ME	SDE	MAE
Fusion of PPG IBI and PPG Morphology	−0.25	17.54	11.16	0.11	9.42	6.60
Fusion of PPG IBI and Calibrated Mathematical Model	0.70	17.22	11.03	−0.03	9.60	6.76
Fusion of PPG IBI, PPG Morphology and Calibrated Mathematical Model	0.58	14.85	8.95	−0.03	8.52	6.09

**Table 3:** Blood pressure estimation performance using IBI features in combination with: (1) Estimation from morphology features; (2) Estimation from calibrated mathematical model; and (3) Estimation from morphology features and estimation from calibrated mathematical model from 30 PPG waveforms. Results are averaged over all the patients.

	Systolic BP (mmHg)			Diastolic BP (mmHg)		
	ME	SDE	MAE	ME	SDE	MAE
PPG IBI and PPG Morphology	−1.51	11.23	7.50	−0.42	6.14	4.94
PPG IBI and Calibrated Mathematical Model	2.52	12.15	8.89	0.59	7.07	4.92
PPG IBI, PPG Morphology and Calibrated Mathematical Model	−1.15	10.69	7.41	−1.11	6.07	4.90

**Table 4:** Correlation between blood pressure estimation using three different methods. In the table, IBI refers to BP estimation using PPG dynamics, morphology refers to BP estimation using PPG morphology features and calibration refers to BP estimation using the calibrated mathematical model. All three methods are described in the text and the results are from 30 PPG waveforms.

Correlation between BP Estimation Using Different Methods for PPG Signals			
	IBI and Calibration	IBI and Morphology	Morphology and Calibration
Systolic BP	0.23	0.16	0.74
Diastolic BP	0.45	0.29	0.78

**Table 5:** Blood pressure estimation performance using PPG signals from 200 patients. The estimation is based on: (1) IBI features; (2) Morphology features using feedforward artificial neural network; (3) Morphology features using feedforward deep artificial neural network; (4) Morphology features using LTSM; (5) Morphology features using GRU; (6) Combining IBI features and estimated BP values from feedforward artificial neural network using morphology features; (7) Combining IBI features and estimated BP values from feedforward deep neural network using morphology features; (8) Combining IBI features and estimated BP values from LSTM using morphology features; and (9) Combining IBI features and estimated BP values from GRU using morphology features.

	Systolic BP (mmHg)			Diastolic BP (mmHg)		
	ME	SDE	MAE	ME	SDE	MAE
PPG IBI	0.09	18.81	14.49	0.03	7.91	5.75
PPG Morphology using Feedforward Neural Network Model	−0.52	20.30	14.51	0.64	9.29	6.78
PPG Morphology using Feedforward Deep Neural Network Model	0.36	13.81	11.24	0.12	6.49	4.75
PPG Morphology using LTSM Model	−0.17	19.11	15.20	−0.05	7.35	5.59
PPG Morphology using GRU Model	0.07	19.22	15.30	0.10	7.29	5.59
PPG Morphology using Feedforward NN Model and PPG IBI	0.01	16.38	13.04	−0.30	6.67	5.31
PPG Morphology using Feedforward Deep NN Model and PPG IBI	0.15	12.40	9.74	−0.01	6.29	4.65
PPG Morphology using LSTM Model and PPG IBI	0.11	18.49	14.63	−0.04	7.77	6.05
PPG Morphology using GRU Model and PPG IBI	0.10	18.87	14.90	−0.04	7.78	6.07

**Table 6:** Blood pressure estimation performance using the proposed model on the test data from 25 never-seen-before patients.

IBI and Estimation from Feedforward Deep NN with ANN Model			
	ME (mmHg)	SDE (mmHg)	MAE (mmHg)
Systolic BP	−4.02	10.40	7.41
Diastolic BP	−0.31	4.89	3.32

## Data Availability

In this study, the bio-signal data from the University of Queensland Vital Signs Dataset [27] and the University of California Irvine Machine Learning Repository [28] are used.
